# Decision support in the management of musculoskeletal disorders: a qualitative study of physiotherapists’ and patients’ experiences

**DOI:** 10.1186/s12891-026-10076-9

**Published:** 2026-06-24

**Authors:** Nina Elisabeth Klevanger, Anita Formo Bones, Ingebrigt Meisingset, Fredrik Granviken

**Affiliations:** 1 Department of Public Health and Nursing, Faculty of Medicine and Health Sciences, Norwegian University of Science and Technology (NTNU), Postboks 8905, MTFS, Trondheim, 7491 Norway; 2 Clinic of Rehabilitation, St. Olavs Hospital, Trondheim, Norway; 3 Unit for Physiotherapy Services, Trondheim Municipality, Trondheim, Norway

**Keywords:** Case-based reasoning, Data sovereignty, Clinical reasoning, Clinical autonomy

## Abstract

**Background:**

Clinical decision support systems (CDSSs) present a paradigm shift in health care by assisting complex decision-making processes. While implementing such systems has considerable potential, they simultaneously influence clinical encounters by impacting decision-making authority. SupportPrim PT (physiotherapy) is a CDSS that uses artificial intelligence methods to provide personalised treatment recommendations to patients with musculoskeletal disorders based on similar successful patients. This study aimed to explore how the CDSS influences the decision-making process of physiotherapists and patients.

**Methods:**

This qualitative study is based on individual interviews with 11 patients with musculoskeletal disorders and 10 physiotherapists in Norwegian primary healthcare in 2021 who have used the SupportPrim PT CDSS. The interviews were analysed thematically, with a theoretical approach inspired by Actor-Network Theory.

**Results:**

First, both patients and physiotherapists valued the visualisation of patients’ biopsychosocial health status. It helped address health issues and expand and align perceptions of relevant information, leading to trust in physiotherapists’ clinical reasoning. Nevertheless, revealing health problems also occasionally created insecurity. Second, the physiotherapists mainly felt that the CDSS treatment decision screen supported their reasoning. For some, it served as a pedagogic tool, lending the physiotherapist authority by confirming the accuracy of their treatment suggestions. However, many found it redundant to use with patients, as they felt secure in how to proceed. Patient involvement was also challenged by occasional inappropriate treatment suggestions and abundant and complex information, diminishing therapists’ trust in the CDSS.

**Conclusion:**

CDSSs can prove valuable by cultivating trust in physiotherapists’ clinical reasoning. However, this trust can simultaneously challenge shared decision-making with the patient. Other aspects influencing decision-making include expectations of the CDSS, its timing, and the content of treatment suggestions. The physiotherapists’ ability and need to interpose between patients and the CDSS to counter inappropriate information also impede shared use. Future studies should investigate how such CDSSs can enhance patient agency by allowing access and addressing the changing role of the physiotherapist once a CDSS is introduced in the clinical encounter.

**Supplementary Information:**

The online version contains supplementary material available at https://doi.org/10.1186/s12891-026-10076-9.

## Background

Clinical decision support systems (CDSSs) are increasingly used to assist in the complex decision-making processes of clinicians and patients and are described as a paradigm shift in present-day healthcare [1]. Existing in various forms to suit a multiplicity of purposes, CDSSs generally pair individual patient characteristics to a computerised knowledge base and generate assessments and treatment recommendations through software algorithms [2]. CDSSs have many benefits, such as providing diagnostic- and patient-facing decision support, increasing patient safety and clinical management [1], improving outcomes, and widening the scope of practice [3]. However, they are also found to cause fragmented workflows and alert fatigue, depend on computer literacy, negatively impact user skills, require maintenance, and lack transportability and interoperability with other systems [1]. In addition, healthcare personnel may find CDSSs insufficient to embed in practice and feel uncertain regarding their mechanics and skeptical of their ability to inform clinical decisions [3].

A variety of factors influence the uptake of CDSSs. While most studies focus on usability [4], uptake is also found to be best predicted by context and implementation strategy [5]. CDSSs are not merely computer technology; they are intimately connected to sets of practices contained by networks of actors situated in their specific contexts [6]. The implementation of CDSSs is a process consisting of multiple stakeholders with varying perceptions [3, 7]. However, whereas healthcare personnel’s perspectives are well represented, the perspectives of other relevant actors, such as patients, are lacking, limiting the understanding and management of such CDSSs [3]. Thus, it is necessary to gain knowledge of how they are used in various contexts and for various patient groups.

Musculoskeletal (MSK) disorders are the leading cause of years lived with disability worldwide and represent a considerable burden to the individual and society [8]. Clinical decision support tools for this patient group are rapidly expanding; however, inconsistent or preliminary findings with limited validity testing, effectiveness- and impact evaluation studies have been reported [9–13]. The SupportPrim PT (physiotherapy) is a CDSS developed to provide biopsychosocial treatment suggestions for managing musculoskeletal pain disorders in primary care [14]. The CDSS identifies similar successful patients and their treatment and aims to support the shared decision-making process of physiotherapists and patients. In physiotherapeutic care, shared decision-making can be defined as a process that “enables a clinician and patient to participate jointly in making a health decision, having discussed the options and their benefits and harms, and having considered the patient’s values, preferences and circumstances” [15], p. 35. However, introducing CDSSs presents a paradigmatic shift from a dyadic relationship between the patient and the clinician into a triad, including the CDSS [16]. Whereas CDSSs have been found to align agendas and empower patients to present their preferences more efficiently [3], they may also introduce several challenges in patient‒clinician interactions. For example, they can lead to changes in responsibility and decision-making authority [17], and some healthcare workers fear that professional autonomy will be reduced [4].

A qualitative study exploring the development and acceptance of a preliminary version of the SupportPrim PT CDSS found that while it positively impacted the therapeutic relationship, patients were seldom included in treatment decisions [18]. However, the study did not explore decision-making dynamics specifically, and the physiotherapists were not experienced in using the CDSS. Therefore, this study aims to explore how a CDSS providing personalised treatment support for MSK disorders influences the decision-making process of physiotherapists and patients.

## Methods

### Study design and setting

This qualitative study is based on individual interviews with physiotherapists and patients within Norway’s primary healthcare sector. The physiotherapists and patients were recruited from a randomized controlled trial, registered in ISRCTN (ISRCTN17927832, February 18th, 2021), investigating the effectiveness of using the SupportPrim PT CDSS compared to usual care [19]. The trial included 44 physiotherapists from 33 physiotherapy practices across Norway, and patients booking appointments were asked whether they could be contacted by a researcher about participating in the study. The results of the clinical trial are reported elsewhere [19].

In Norway, patients have direct access to physiotherapy in primary health care. The first consultation usually lasts 30–60 min, whereas the second consultation lasts between 20 and 30 min, and treatment commonly consists of a combination of education, exercises, and passive modalities. The SupportPrim PT CDSS (hereafter referred to as `the CDSS´) was developed to improve healthcare management of common MSK disorders in primary physiotherapy care [14]. Since these disorders are highly individual and influenced by a variety of biological, psychological, and social factors, the CDSS aims to account for patients’ heterogeneity by providing personalised treatment. Using case-based reasoning [14], a problem-solving method in artificial intelligence, the CDSS identified similar patients and their treatment, intending to help patients and physiotherapists decide on appropriate treatment through shared decision-making. To prevent recommendations from being driven solely by past cases, the CDSS also incorporated evidence-based guidance aligned with clinical guidelines. This included advice targeting modifiable biopsychosocial factors (e.g., physical activity, sleep, stress, fear avoidance, self-efficacy) and general management suggestions informed by the patient’s prognostic risk profile.

The SupportPrim PT CDSS intervention was carried out as follows. The physiotherapists (hereafter referred to as `therapists´) received educational material and individual education on how to use it. Patients answered web-based questionnaires before attending their first consultation, and the answers were displayed on a patient profile screen (Appendix 1). The therapists could access this profile before the first consultation. The CDSS was designed for patients and therapists to explore together, before defining treatment goals. The therapists also needed to perform an examination and enter a diagnosis for the CDSS to identify the three most similar previous patients with successful outcomes, defined by a composite score of biopsychosocial domains [14]. The patient and the three most similar patients were then displayed on a treatment decision screen (Appendix 2), which also included personalised treatment recommendations based on factors associated with high symptom severity (such as low physical or social activity, sleeping disorders, mental distress, poor workability, fear avoidance, low pain self-efficacy, and low expectations of recovery). The CDSS also contained online resources with printable links related to the patient’s condition, based on self-reported factors such as sleep problems that may be targets for treatment. Detailed descriptions of the development and content of the CDSS are published elsewhere [14, 18].

### Research participants and recruitment

This study is based on a purposive sample of participants. Ten therapists and 11 patients who participated in the randomised controlled trial were recruited by email (therapists) or telephone (patients) by AFB or NEK for individual, semi-structured interviews conducted between April and June 2021. Therapists were primarily recruited based on their experience with using the CDSS, and we also aimed to include participants presenting a variety of sexes, agees, clinical experience, and specialities. Seven therapists were general physiotherapists, three had education in manual therapy, and one was educated in psychologically informed physiotherapy (Table 1).

**Table 1:** Physiotherapist characteristics

Number	Age-range	Sex*	Clinical experience (years)	CDSS use (no. of patients)
1	30–39	M	15	12
2	20–29	M	7	8
3	40–49	M	24	8
4	40–49	F	21	11
5	30–39	F	16	8
6	30–39	M	10	12
7	40–49	M	17	7
8	40–49	F	18	14
9	40–49	F	16	8
10	30–39	F	12	14

*M male, *F *female

The inclusion criteria of the RCT were broad to reflect patient heterogeneity in current physiotherapy practice; patients aged 18 years and older with clinician-determined MSK pain in the shoulder, neck, upper/low back, hip, knee, or with multisite/complex pain of any duration as the main reason for seeking physiotherapy were included. Exclusion criteria were reduced cognitive function, insufficient Norwegian language skills, ongoing cancer, pregnancy or pregnancy-related conditions, surgery or fracture within the past 6 months, scheduled surgery, or a neurological diagnosis [19]. The patients were consecutively recruited based on the number of times their therapist had used the CDSS. To ensure adequate user experience, we contacted patients whose therapists had used it with at least six previous patients before their first consultation. Five patients did not reply to our invitation to participate. We discontinued recruitment once the interviews yielded little new insight in terms of depth and variation of experiences. The interviews were conducted within two months of the first consultation (Table 2).

**Table 2:** Patient characteristics

Number	Age-range	Sex*	Main problem	Number of pain sites	Pain duration (months)
1	20–29	F	Knee	-	1–3
2	30–39	F	Multisite/complex	7	6–12
3	20–29	F	Neck	4	1–3
4	50–59	M	Shoulder	4	> 12
5	40–49	F	Hip	3	< 1
6	30–39	F	Multisite/complex	-	< 1
7	20–29	M	Back	2	> 12
8	50–59	F	Knee	3	6–12
9	20–29	M	Back	1	> 12
10	20–29	F	Multisite/complex	10	> 12
11	50–59	M	Knee	1	1–3

*F female, *M *male

Both interview guides were developed for this particular study. The interview guide for the therapists (Appendix 3) included questions regarding their approach when using the CDSS, what they found valuable and challenging, what changes they had to make to ordinary practice, who they believed would benefit from using the CDSS, and their perceptions of how patients experienced using it. The interviews increasingly focused on exploring the therapists’ reasoning on how they used the CDSS with specific patients and chose not to use it with others. The interview guide for the patients (Appendix 4) included questions regarding their experiences with using the CDSS, for example, if the patient profile reflected their understanding of their health and how they felt about the therapists having access to it before the first consultation, the relevance of using similar patients to decide on treatment, what they found valuable and how the CDSS could be changed to better suit their needs. The interviews increasingly focused on exploring whether patients felt involved by their physiotherapists and how they interacted in using the various parts of the CDSS, especially when deciding on treatment.

### Data collection

The participants were asked if they wanted to be interviewed by telephone or in a digital meeting since they resided in various parts of the country. All interviews lasted approximately 30–60 min, and the audio was recorded and transcribed verbatim. Either NEK or NEK and AFB together performed the interviews, both of whom had previous experience conducting interviews.

### Theoretical approach

Actor-Network Theory [20, 21] has inspired both the analysis and the discussion of the results. Viewing the social and technological dimensions as intimately entwined, the theory has been described as useful for exploring how technology shapes social processes [22–24]. Actor-Network Theory focuses on the interplay between technology and its users, seeing nonhuman elements as actors that can enable and mediate action by interacting with other actors. The reciprocal relationship between the technological and the social is further described by the term *delegation* [20], while we delegate work to technologies, technologies also delegate behaviour back into the social, influencing and shaping actions within sociotechnological networks. As such, the theory is not concerned with merely mapping interactions; it includes the process of *translation* [21] in which the actors define roles and delimit and negotiate the possibility of interaction and their margins of manoeuvre [21]. In this study, Actor-Network Theory is used as a framework to explore how patients and therapists interact with each other and with the CDSS and how these interactions influence the decision-making process.

### Analysis

The interviews were analysed thematically [25], inspired by the Actor-Network Theory perspectives on interaction [20, 21]. One researcher with a social science/public health research background (NEK) and three with physiotherapy and public health research backgrounds (FG, AFB, IM) contributed to the analysis. NEK read all the interviews, noting initial thoughts on their content before coding them individually. FG and AFB read and coded two interviews to compare interpretations. The interviews were coded inductively, paying particular attention to how the CDSS influenced experiences and interactions during the decision-making process. In line with the theoretical approach, we also attempted to identify occasions where the participants described how the CDSS influenced their thoughts, feelings, or actions through the following question: `What does the CDSS *do* and *how*?´. As such, the themes are analytic abstractions of participants’ descriptions of their experiences using the CDSS and what they believed it contributed to. Patient interviews revealed that few had experience with the treatment decision screen. Although additional interviews might have yielded additional details, the consistent pattern observed simultaneously constitutes a key finding of the study. Consequently, the themes related to this part of the CDSS are informed predominantly by therapist interviews. Divergent perspectives were examined systematically to ensure that variations in experience were adequately represented. NEK, FG, and AFB discussed the coding and preliminary themes during several meetings and developed initial themes including visualisation, lending authority, and aligning perceptions of health. Then the first draft of the results was prepared and discussed with the remaining author IM. During the subsequent analytic process, the themes were adjusted into a chronological narrative based on how the CDSS was used. These are presented in Table 3 as two main themes corresponding to the CDSS´s structure. Quotes were selected to provide an interpretational context and exemplify or nuance the analytic text.

**Table 3:** Coding tree

Main themes, sub themes and quotes
1 Visualising health status
◦ Expanding and aligning perceptions of health issues “We will perhaps talk about more than *just* the painful knee”
◦ Providing latitude to address health issues “I find it a lot easier to point things out”
◦ Creating insecurity “It is perhaps almost a bit intimidating for those with many and complex issues”
2 Confirming treatment decisions
◦ Supporting clinical reasoning “I feel that I get confirmation on doing a lot that others are doing too”
◦ Lending authority to therapists “`That´s exactly what we agreed upon last time, then we know we are on the right track´”
◦ Demanding active management “What factors does the system use to find these similar patients?”

### Ethical considerations

The Regional Committee for Medical and Health Research Ethics in Norway approved the study (49308/2020), and all patients provided written informed consent before participating. The study was performed in accordance with the Declaration of Helsinki. Since the therapists and patients were not matched, the interviews with the therapists did not reveal the health information of identified patients. To ensure anonymity, age is described in ranges for all participants, and the speciality of individual therapists is omitted.

## Results

The interviews showed that the two main parts of the CDSS influenced the decision-making process differently. Therapists and patients described the visualisation in the patient profile as particularly influential in the initial process, as it expanded and aligned perceptions of health issues and provided latitude to address a broader range of concerns within a process marked by high patient involvement. However, it could also create insecurity when sensitive topics were introduced. Whereas therapists appreciated how the CDSS predominantly confirmed their treatment decisions, the patient interviews revealed that only two patients had used the treatment decision screen with the therapist, and six had not seen any screens. The following results expand on how these findings influenced the decision-making process.

### Visualising health status

#### Expanding and aligning perceptions of health issues

Patients and therapists described the CDSS as enabling them to expand and align their understanding of the patient’s health issues and treatment needs. Several patients said they would have revealed less information without the CDSS:

Otherwise, I don´t talk much about my life; I´m not the type who says much. (…) Therefore, the questions regarding sleep and such things were very accurate in my case because I struggle to sleep. (Patient 7)

The therapists found that answering the questionnaires primed patients into a psychosocial view of health and made them better prepared for the first consultation, which improved consultation flow and facilitated their work.

The patients are somewhat more prepared for what will happen—that we will perhaps talk about more than *just* the painful knee when they enter the door. And they have thought about the bigger picture and how it affects their life before they arrive. (Therapist 9)

Although the therapists said they retrieved similar information to that displayed in the patient profile in their practice, it usually required several consultations. Some also said that the CDSS revealed information they did not address in their regular anamnesis. For example, one therapist had become attentive to how she often refrained from addressing psychosocial issues with some men, and that the CDSS provided surprising information:

I believe patients communicate easily with me, and now I understand that if it wasn´t for this [the CDSS], there are things I probably would have missed” (Therapist 8).

As such, the CDSS revealed information that also reminded therapists to pay increased attention to psychosocial health aspects.

#### Providing latitude to address health issues

Both therapists and patients highly valued the CDSS for providing a thorough first impression of the patient’s health status, which helped them address relevant issues. The patients predominantly found that the patient profile reflected their health experiences and gave therapists an improved impression of their situation. One patient described the visualisation in the CDSS as an objective representation of her health:

I liked it, truly - to have more perspective on it [my health situation], and it’s a good thing that others see it as well; it becomes a bit less subjective. (Patient 1).

The therapists also described how the CDSS provided a more authentic or accurate representation of health that facilitated their approach, as patients were more or less statistically unfolded on the screen:

If psychosocial challenges are showing red, it is much easier to talk to the patient about it and address the various themes. After all, the patients said it themselves, only in advance. (Therapist 3)

As such, the CDSS was found to grant therapists the latitude to address relevant aspects of treatment. Viewing the screen together was described as a valuable means of facilitating `the good conversation´ as it was disarming for patients compared with potentially uncomfortable eye contact in regular consultations.

Since the CDSS revealed comprehensive patient information, the therapists also described feeling obliged to address it. Although the patients predominantly seemed to find it useful, therapists noted that some appeared surprised when issues other than their main concern were introduced:

Last week, I had someone with *poor* mental health (emphasis in original). But she was here because of a knee, so when I addressed it and said: `I can see this [in the CDSS]´, tears were running, and there was a conversation about it and a note to the general practitioner about it. Then, we continued to work on what we do. (Therapist 8)

#### Creating insecurity

Although the patients underlined the importance of addressing relevant health issues, sharing and addressing sensitive information could be demanding. For instance, one patient said that he felt intimidated by facing the therapist after sharing information about his mental health. The therapists also believed that the visualisation of health information and prognosis could be discouraging for some, making it necessary for them to view the patient profile beforehand, proceed cautiously, and downplay alarming information:

I have experienced that it is perhaps almost a bit intimidating for those with many and complex issues. Especially this first picture, where every arrow is red, it can be a bit… Well, I have been unsure to what extent I want to view it [with the patient] simply because it lights up red in every corner and looks dismal. (Therapist 8)

It was also noted that being made aware of high symptom severity before meeting the patient could negatively influence therapist assessments.

While patients underlined that they were aware of their health issues beforehand, some became more attentive to their relevance or scope after having them visualised:

I started to reflect more about my sleep because it… I do not believe I reflected upon answering that it was poor, but this system picked up moderate or medium sleep problems. So that was more evident to me. (Patient 1)

One therapist noted that comprehensive mapping and visualisation of health issues could lead to overtreatment, as patients are perhaps satisfied with merely a few practical tips. Having to attend to sensitive information was also described as occasionally challenging, particularly by therapists who were left to attend to the patient’s issues themselves as they were practising in high-pressure or rural settings with few referral opportunities. Nevertheless, both therapists and patients consistently emphasized that providing relevant information was beneficial for addressing key treatment factors.

### Confirming treatment decisions

#### Supporting therapists’ reasoning

When explaining what a *decision support system* entailed in this setting, therapists commonly underlined that the role of the CDSS was to support them in deciding on appropriate treatment. Although a few described utilising some treatment suggestions from the CDSS, most said it supported already planned treatment. This was particularly valuable due to their work context:

I use it as a support, a backing actor in a way. And to me, who is without colleagues, situated alone in the outback, it is worth its weight in gold (…) I feel that I get confirmation on doing a lot that others are doing too, and you might get some useful suggestions as well. (Therapist 4)

Although involving patients was described as essential to provide ownership of the treatment process, the therapists usually referred to patients as `more active than usual´ when using the patient profile. However, the CDSS was seldom referred to as enabling shared decision-making regarding treatment, and the therapists described that not all patients should be included if the therapist felt secure in their treatment choice. Patient interviews mirrored this perspective; they told of feeling very much included in the consultations, while most were happy to leave the task of deciding on treatment to the therapist:

I was like: `Just give me exercises I can do or give me a plan, and I will follow it´. (…) I do not have the knowledge about that, and then I trust those who know more” (Patient 1).

However, the therapists also addressed the need to ensure that the discrepancy between patients´ wishes and what therapists considered appropriate treatment was not too large to maintain trust, and some told of occasionally providing treatment they found unnecessary for this reason.

#### Lending authority to therapists

In addition to providing confidence in their reasoning, therapists spoke of using the CDSS treatment decision screen as a pedagogic tool. They showed it to patients when it confirmed their treatment plans, and described that it enabled them to build trust, reason with, or persuade patients. Reflecting patients’ descriptions of the CDSS as a more objective representation of health, therapists found it useful in helping determine what is `true´:

A nice thing about this system is that you get a clarification of what is true, right, it isn´t just me, I have backing for what I am saying. (Therapist 6).

As such, the CDSS can be said to lend the therapist authority—if their suggested treatment aligned with the CDSS. In addition, reviewing the graphs explaining progression over time was described as an aid that could normalise an initial increase or slow decrease in pain and thus reassure patients and build motivation.

#### Demanding active management

Although therapists mainly described the treatment suggestions as valuable confirmations, they occasionally found them inappropriate and deviating from their clinical reasoning. They also considered the information in the treatment decision screen to be abundant and often too complex for patients, requiring active management when choosing to show it. The therapists sometimes questioned the CDSS’s algorithms when finding patients identified as similar by the CDSS inappropriate:

What factors does the system use to find these similar patients? What are they comparing? Sometimes it is very evident, other times I am thinking: `these patients are just entered randomly and doesn´t fit at all´” (Therapist 9).

Low trust in the CDSS’s suggestions of similar patients was attributed to the small patient pool, and when encountering dissimilar suggestions, therapists told of avoiding showing them to patients. In other cases, seemingly similar patients had unsuitable treatment suggestions, for instance, for some patients diagnosed with chronic fatigue syndrome, they were pointed out as contraindicated owing to the proposed activity level. The therapists also experienced that the CDSS could suggest treatment supporting patients’ biomechanical view of health or wish for passive treatment:

We have perhaps started talking about a plan, using more active management strategies, exercises, and have a comprehension of how to handle the pain [after using the patient profile]. And *then*, when we open the part with similar patients, and it describes that it is very useful to receive stretching and passive treatment (laughs). When I see that, I do not want to open that door when we have already started moving in another direction. (Therapist 2)

As the patient profile was commonly viewed as sufficient to set a treatment course, the treatment decision screen could disrupt practice flow if suggestions diverged. Therapists usually waited to use it until the second consultation due to the additional time required to review the patient profile, uncertainty about treatment suggestions, and the overall complexity of this CDSS part. In contrast to the simplicity of the patient profile, the treatment decision screen was described as challenging because it required the therapist to provide thorough explanations. Whereas the therapists described feeling confident in using the CDSS after 3–4 patients, some were still unsure in utilising this part of the CDSS optimally after ten or more, and described that they sometimes avoided showing it altogether to prevent insecurity.

## Discussion

This is the first study to explore experiences with a CDSS based on case-based reasoning providing personalised treatment suggestions for MSK disorders. We found that patients and therapists valued the visualisation of health status, as it provided latitude to address relevant health aspects and align views, although occasionally creating insecurity. Therapists felt supported by the CDSS since the treatment decision screen largely reflected their clinical reasoning, and they sometimes used it to lend authority. However, the CDSS occasionally contained complex information and inappropriate treatment suggestions, leading therapists to share them selectively. Patient interviews revealed that few had seen the treatment decision screen, reflecting their lack of involvement in shared decision-making.

### The dynamics of the decision-making process

Whereas CDSSs are previously found to facilitate shared decision-making [3, 26], this study sheds light on how and why therapists may adjust patient involvement based on the perceived appropriateness of CDSS information. The findings largely align with previous research showing that the SupportPrim PT CDSS was valued as a preparatory and exploratory tool strengthening the therapeutic relationship, whereas treatment suggestions mainly supported ordinary practice rather than involving patients [18]. Although therapists´ limited CDSS experience could explain low patient involvement, the present study reveals that more experienced therapists often chose not to as well. Inspired by the Actor-Network Theory concept of *translation*, a process where “the identity of actors, the possibility of interaction and the margins of manoeuvre are negotiated and delimited” [21], p. 201, the following discussion explores how the CDSS, therapists, and patients influence one another´s actions throughout the decision-making process. We explore these dynamics through the CDSS´s main parts: (1) the patient profile, where health problems are defined, and (2) the treatment decision screen, where treatment is suggested.

1) Defining the problem

The results suggest that the CDSS plays an important role in shaping views on the patient’s health problem. It can be said to *delegate* [21] the behaviour of other actors by determining questionnaire topics and transforming the answers using inherent cut-offs in the patient profile, thereby shaping how to proceed. A main finding is that thorough patient mapping and shared viewing of the patient profile predominantly increased trust - both patients’ trust in therapists and therapists’ trust in their own reasoning. Decision-making includes the role of patients´ illness narratives in relation to CDSS information, and previous studies have found that clinicians´ trust in CDSSs decreases when clinical detail is lacking [26]. In the present study, therapists found that the thorough patient profile information exceeded their regular anamnesis, providing a sound basis for subsequent decision-making. However, it occasionally included information beyond their qualifications, and patients seeking knee injury advice could unexpectedly find themselves discussing mental health issues. Thus, employing CDSSs may deviate from dealing with patients´ concerns by conveying secondary findings, and the ethical aspects of physicians addressing issues beyond their competency have been linked to medicalisation [27]. A previous study has also found that Norwegian physiotherapists believe integrating psychosocial domains depends on clinical experience and may exceed their professional competence [28]. Therefore, education on how to address the scope of patient information revealed by the CDSS is essential, especially since patients and therapists tend to view it as more `objective´ or `true´ than patients´ subjective accounts.

From an Actor-Network Theory perspective, the patient profile facilitated enrolment into a shared problem framing, giving patients and therapists latitude to address relevant topics and align perceptions. At the same time, it broadened problem definition in ways that could create insecurity and unsettle roles and boundaries. These dynamics show how the CDSS helped assemble a network in which identities and authority were redistributed, shaping the conditions under which shared decision-making could later unfold.

2) Deciding treatment

Although the patient profile predominantly increased trust and patient involvement, it nevertheless appeared to impact the use of the treatment decision screen negatively, as few patients had seen it. This underscores the need to analytically distinguish patients’ subjective sense of involvement from the formal practice of shared decision-making the CDSS was designed to support. While our understanding of this CDSS part relies on therapists’ accounts, we identified several factors that may explain why shared decision-making was not performed as intended. 

First, patients’ and therapists’ *expectations of the CDSS and each other’s roles* seemed to influence CDSS use. Therapists often interpreted `decision support´ as support for their own reasoning rather than viewing the CDSS as a tool for shared decision-making, suggesting insufficient terminology and training. Although patients described their involvement as `high´, this mainly reflected feeling more involved than expected in discussing health issues and did not translate into a preference to choose treatment. This reflects previous findings that patients want to keep clinicians in the decision-making loop due to their clinical experience and ability to evaluate CDSS outputs [29]. While shared decision-making entails the patient’s right to participate to the extent they want [30], it is essential that users understand the CDSS´s purpose and potential. When generating treatment suggestions, the CDSS did not explicitly incorporate patient values and preferences, key components of shared decision-making [15]. Moreover, dynamic variables such as behaviours and environment are seldom incorporated into CDSS algorithms [31], and patients´ autonomy and treatment authority can be undermined unless therapists actively integrate them. This underlines the need for therapists to consider the broader patient–therapist–CDSS context, including how patients’ expectations for involvement affect shared decision-making.

Second, the *timing and content of treatment suggestions* appear to reduce the CDSS´s relevance to shared decision-making. Therapists considered the patient profile sufficient for initiating treatment, whereas the treatment decision screen was too time-consuming for the first consultation and disruptive if introduced later. This is a key limitation, since CDSSs providing support at the time and location of decision-making succeed better [32]. The recommendations also largely confirmed therapists’ reasoning, suggesting the CDSS aligned with established practices by presenting a logic of continuation rather than disruption or change [33], which was the CDSS´s intention. Moreover, the recommendations were perhaps insufficiently tailored to the substantial patient heterogeneity of physiotherapy practice. An important limitation of the CDSS was that it matched patients using case-based reasoning based on prognostic factors, identifying similar patients rather than prescribing specific treatments. As such, treatment suggestions reflected what had been provided to previous patients with successful outcomes without any built-in control of treatment modality, although the CDSS also included evidence-based guidance and clinical guidelines to prevent recommendations from being driven solely by past cases. As innovations positively impacting clinical routines by adding meaning are more likely to succeed [34], the limited value in informing treatment decisions is a significant limitation to the CDSS´s intended use as a shared decision-making tool.

Third, patient involvement in shared decision-making was also limited by the *therapist’s ability and need to interpose* between the CDSS and the patient. Whereas this may avoid confusion and build trust, it simultaneously appeared to reduce patients’ agency in making treatment decisions, a contradiction previously described in a study of Swedish physiotherapy patients’ preferences for treatment and participation in decision-making [35]. Furthermore, a CDSS´s lack of transparency and clinicians’ superior understanding of them may restrict patients´ ability to act as informed participants in shared decision-making [29]. This asymmetric agency enabled therapists to use the present CDSS as a source of authority, ensuring that their own treatment suggestions appeared valid. Whereas physiotherapy patients are found to have great confidence in therapists´ skills [35], CDSSs may alter clinicians’ decisional authority by influencing expectations, verdicts, roles, and responsibilities [29]. Clinicians’ fear of losing control over autonomous decision-making and having clinical autonomy reduced is identified as the most severe barrier to CDSS uptake [4]. Although such concerns may have discouraged therapists from using treatment suggestions as intended, the abundant and complex information of the present CDSS also caused interpretative flexibility, *requiring* them to interpose. Thus, therapists experienced with the CDSS may have found it redundant in straightforward cases and unsettling when cases were severe or inappropriate recommendations appeared. The value of individual data sovereignty, where patients can exercise informational self-determination by co-managing their data, is highlighted in the context of CDSSs [29]. Whereas such access could enhance shared decision-making within the present CDSS, it also removes therapists´ ability to screen information, emphasising the need for high-quality, relevant output.

From an Actor-Network Theory perspective, the CDSS positioned therapists as obligatory passage points controlling how outputs were interpreted, filtered, or withheld. This created asymmetric agency, as therapists could mobilise the CDSS to support their own reasoning and thereby lend authority. If treatment suggestions offered little novelty, the CDSS acted as a stabilizing rather than transformative actor, and therapists’ centrality was further reinforced when information opened up for interpretative flexibility. These dynamics reveal how the CDSS reconfigured relations around treatment decisions without redistributing decisional authority as intended. Supporting clinicians in navigating these altered relations and ensuring that treatment suggestions appear meaningful is essential if CDSSs are to enable rather than constrain shared decision-making.

### Strengths and limitations

This study contributes valuable information on the mechanisms of using a CDSS, i.e., how various aspects of a system might be experienced as meaningful by users while still undermining its intention. The inclusion of both patients and therapists increases the credibility of the findings. Also, authors represent various backgrounds, and the study results have been presented and discussed in research groups consisting of participants with knowledge of both the topic, patient group, and methodology. Whereas authors IM, FG, and AFB developed and implemented the CDSS, and FB and AFB educated the therapists on how to use the CDSS, the first author, NEK, contributed to the evaluation process. Physiotherapists with various specialities participated in the study. While the findings suggest some variation between the experiences of the physiotherapist with education in psychologically informed physiotherapy and the others, we chose not to investigate this further because only one therapist with such education was recruited. The patients’ interviews did not provide sufficient knowledge to investigate their experiences with the treatment decision screen in detail, and the results lean heavily on the therapists’ experiences with this part of the CDSS. Although recruiting more patients could have yielded more information, the low number familiar with this part of the CDSS is also a key finding of the study. We therefore chose to focus on the therapists´ reasoning for not including patients when using it. A strength of the study is the extensive participant quotations, which enhance qualitative rigor by providing thick description, preserving participants’ voices, and making the analytic process more transparent. These excerpts allow readers to engage directly with the nuance of participants’ accounts, thereby strengthening the credibility and confirmability of the findings. Special attention was given to diverging perspectives, which were systematically explored and incorporated to enhance the depth and credibility of the findings. We have also provided a detailed description of the study setting, participants, and analytic procedures to support transferability and strengthen dependability. A limitation of the study is that the interviews relied in part on more focused questions to explore the connection between specific CDSS components and shared decision-making. This may have constrained participants’ narratives, and more open-ended prompts could have enabled them to elaborate more freely and produce richer, more nuanced insights. The majority of the interviews were conducted by telephone, possibly omitting important nonverbal information. Focus group discussions with therapists could also have yielded further information on their perceptions and reasoning for system use, and observational studies could have provided valuable information on the interaction between the actors and how the therapists differentiated their use according to specific patient characteristics. Future studies would benefit from such methodological triangulation.

## Conclusion

The results of this study provide insight into the dynamics of the decision-making process between physiotherapists and patients when using a CDSS to decide on treatment for MSK disorders. Although restricted patient exposure limits firm conclusions about the CDSSs´ impact on shared decision-making, we found that expanding and aligning views on patients’ health issues through increased patient involvement may cultivate trust in the therapist’s clinical reasoning. However, the study suggests that increased trust may simultaneously impede shared decision-making. Other aspects that may influence decision-making dynamics include expectations of the CDSS and the other actors´ roles, as well as the timing and content of the CDSS. Furthermore, therapists’ ability and need to interpose between patients and the CDSS can reduce patients’ agency, and a lack of joint access allows the CDSS to lend therapists authority instead of enabling shared decision-making. Future studies should investigate how CDSSs can enhance patient agency by providing access and how physiotherapists’ roles change once tools influencing patient interaction and decision-making are introduced into clinical encounters.

## Supplementary Information

Supplementary Material 1.

Supplementary Material 2.

Supplementary Material 3.

Supplementary Material 4.

## Data Availability

The datasets generated and/or analysed during the current study are not publicly available since the study participants’ consent did not include such permission. However, they are available from the corresponding author upon reasonable request and with the permission of the study participants.

## Abbreviations

CDSS: Clinical decision support system
MSK: Musculoskeletal
PT: Physiotherapy
