# Supplementary material for: Decision support in the management of musculoskeletal disorders: a qualitative study of physiotherapists’ and patients’ experiences
Source: BMC Musculoskelet Disord. 2026 Jun 24;27:816. doi: 10.1186/s12891-026-10076-9 (PMC13629069; doi:10.1186/s12891-026-10076-9)
Supplement: Supplementary file 3 [file 12891_2026_10076_MOESM3_ESM.docx]

**Appendix 3**

**Interview guide physiotherapists**

**Introduction**

How many patients have you used the system with?

Do you feel you manage using the system and can provide patients with the necessary information? How many patients did it take to feel you managed using it?

**Approach when using the CDSS**

How many consultations do you need to go through the entire system?

How do you proceed when using the various parts of the system?

• Patient profile

- Do you look at the patient profile before the consultation? How do you feel about having this information before meeting the patient?
- How does the CDSS reflect your assessment of the patients?

• Treatment decision screen

- Do you look at the system´s suggestions of similar patients before showing them to the patient?
- Do you examine the various components with the patient?
- How do you decide on treatment? (E.g. based on what information)

**Shared decision-making/ patient participation**

SupportPrim is a so-called "decision support system". What does that entail to you, and to what means do you use it in your practice?

Does the CDSS influence the dynamics/interaction with the patient compared to your usual consultations? (Does it affect e.g. patient involvement? Your role/status? How do you decide on treatment? Can it lead to disagreements?)

**Use value**

Do you believe this approach to finding a treatment is useful? Why/why not?

- What do you find valuable?
- What do you find challenging or not useful? What could make it more useful to you? What are the potential downsides to using this system in physiotherapeutic care?

What kind of patients and therapists could benefit from using the CDSS? Who would not?

How do you find the information in the CDSS? (E.g. relevancy/transferability, amount, detail, complexity)

What is your perception of how your patients experience using the CDSS?

Would you recommend it to colleagues?

Is there anything I have not asked you that you think is important or that you thought I would ask you?
