# Supplementary material for: Decision support in the management of musculoskeletal disorders: a qualitative study of physiotherapists’ and patients’ experiences
Source: BMC Musculoskelet Disord. 2026 Jun 24;27:816. doi: 10.1186/s12891-026-10076-9 (PMC13629069; doi:10.1186/s12891-026-10076-9)
Supplement: Supplementary file 4 [file 12891_2026_10076_MOESM4_ESM.docx]

**Appendix 4**

**Interview guide patients**

**Introduction**

How many times have you been to the physiotherapist?

Could you briefly explain why you consulted the physiotherapist?

**Approach when using the CDSS**

How did you feel about filling in the questionnaire? (E.g. did it influence your view of your health situation?)

How did you feel about the physiotherapist receiving this information before the first consultation?

How did you and your physiotherapist use the various CDSS parts? What were your experiences of it?

- Patient profile
- How did you experience seeing information about your health presented in this way? (E.g. did it reflect how you feel, or were you surprised by something? Did it influence your experience of your health situation?)
- How did you use the patient profile?
- Treatment decision screen
- Did you look at the various components of the CDSS together?
- How did you choose the most similar patient? Did you feel that it reflected your situation?
- How did you choose treatment? What information influenced the choice? (E.g. information from the CDSS, the physiotherapist's reasoning, your experiences/wishes/motivation)

**Shared decision-making/ patient participation**

How did you experience finding treatment by using the CDSS?

- Did you feel that you were an active part of the consultations? When and how?
- To what extent did you feel that your experiences and wishes were involved/considered?

How did you feel the CDSS affected your communication with the physiotherapist? (e.g. screen use, participation in treatment, potential disagreements)

**Use value**

Do you believe this way of finding treatment is useful? Why/why not?

- What did you find valuable?
- What could be changed to better suit your needs or preferences?
- Is there anyone you think would profit more than others from using such a system to find treatment? For whom is it unsuitable?

Do you feel that you used sufficient time going through the CDSS?

Would you recommend it to friends and family?

Is there anything I have not asked you that you think is important or that you thought I would ask you?
