# Supplementary material for: Decision support in the management of musculoskeletal disorders: a qualitative study of physiotherapists’ and patients’ experiences
Source: BMC Musculoskelet Disord. 2026 Jun 24;27:816. doi: 10.1186/s12891-026-10076-9 (PMC13629069; doi:10.1186/s12891-026-10076-9)
Supplement: Supplementary file 6 [file 12891_2026_10076_MOESM2_ESM.docx]

**Appendix 2**

**Treatment decision screen**
